# The relationship between maternal periodontitis and congenital cytomegalovirus: A hypothetical model and therapeutic implications

**DOI:** 10.1111/prd.12632

**Published:** 2025-06-20

**Authors:** Jørgen Slots

**Affiliations:** ^1^ Division of Periodontology and Diagnostic Sciences University of Southern California School of Dentistry Los Angeles California USA

**Keywords:** cytomegalovirus, non‐syndromic congenital disorders, periodontitis, valacyclovir

## Abstract

**Background:**

The primary goal of periodontology is to prevent tooth loss and reduce the risk of focal infections. Periodontitis lesions can harbor hundreds of thousands of active cytomegaloviruses (virions), which can easily enter the systemic circulation and potentially infect the fetus of a mother with compromised immunity. The healthy, non‐inflamed periodontium contains no PCR‐detectable cytomegalovirus. Maternal cytomegalovirus may be linked to cleft lip and cleft palate, prepubertal and juvenile periodontitis, and systemic diseases.

**Aim:**

This article presents an anti‐cytomegalovirus periodontal therapy aimed at preventing congenital cytomegalovirus disorders.

**Materials and Methods:**

Immunodeficient and periodontitis‐affected women in the pre‐gestational period or the first trimester of pregnancy are prime candidates for periodontal treatment. The periodontal diagnosis and drug treatment ought to be performed by well‐informed dentists (periodontists). Treatment consists of a one‐time valacyclovir regimen (1 g BID on days 1 and 2, and 500 mg BID on days 3–7), subgingival and supragingival ultrasonic scaling using a 0.1%–0.2% sodium hypochlorite cooling solution, individually tailored oral hygiene instructions, and patient‐administered daily subgingival irrigation with a 0.1%–0.2% sodium hypochlorite solution.

**Results:**

Cytomegalovirus in maternal periodontitis likely serves as a critical nidus for fetal infection. The combined treatment of valacyclovir, ultrasonic scaling, and sodium hypochlorite rinses markedly reduces or eliminates the mother’s periodontal cytomegaloviruses.

**Clinical relevance:**

The proposed noninvasive anti‐cytomegalovirus periodontal therapy is highly safe for the pregnant mother and the fetus. The anti‐cytomegalovirus periodontal treatment is expected to control the mother’s periodontal cytomegalovirus load and, consequently, part of the baby’s congenital disease risk. Research is encouraged on the relationship between periodontal cytomegalovirus and congenital diseases.

## 1 CYTOMEGALOVIRUS AND CONGENITAL DISEASES

Cytomegalovirus is an enveloped double‐stranded DNA virus that belongs to the family Orthoherpesviridae, the subfamily Betaherpesvirinae, and the human species Cytomegalovirus humanbeta5. It is the largest human herpesvirus, measuring 220 nm in diameter, and encodes more than 150 proteins and 25 microRNAs. Cytomegalovirus sheds into bodily fluids such as saliva, urine, breastmilk, and semen, infects individuals of all ages, can reinfect with immunological variants, provides incomplete protection despite acquired immunity with some risk of reactivation, and remains in the infected body for a lifetime.^1^ Cytomegalovirus can cause severe diseases in unborn babies and individuals with weakened immune systems.^1^, ^2^ In 2011, the US Congress passed a resolution designating June as “National Cytomegalovirus Awareness Month” to increase understanding of congenital cytomegalovirus diseases among healthcare providers, pregnant individuals, and parents.

In the United States, cytomegalovirus has infected nearly 1 in 3 children by age 5, over half of adults by age 40, and almost 90% by age 80.^1^ The virus in healthy immunocompetent individuals typically shows no signs or symptoms or only mild illnesses such as fever, sore throat, muscle pain, and fatigue. However, cytomegalovirus may occasionally cause mononucleosis and hepatitis. In immunosuppressed individuals (HIV/AIDS, cancer chemotherapy), cytomegalovirus may cause persistent oral ulcers and serious illnesses affecting the eyes, lungs, liver, esophagus, stomach, brain, and intestines. Patients receiving organ or stem cell transplants are typically prescribed immunosuppressant medications and may experience cytomegalovirus reactivation, graft‐versus‐host disease, and transplant rejection.

The total estimated number of pregnancies in the United States was 5 507 000 in 2019.^3^ Each pregnancy has a 3%–5% risk of developing birth defects and long‐term health problems.^1^ A pregnant woman may transmit cytomegalovirus virions across the placenta, and congenital cytomegalovirus infection is the most common infectious cause of birth defects. One in 100 women contracts a cytomegalovirus primary/active infection during pregnancy, 1 in 200 newborn babies shows congenital cytomegalovirus infection (cytomegalovirus DNA in the newborn's urine or saliva within 2–3 weeks of life), and 1 in 5 infants (5500 US infants annually) born with congenital cytomegalovirus infection develops health issues at birth or later in life.^1^ Cytomegalovirus infection during the preconception period or the first trimester of pregnancy may result in physical or neurologic sequelae in 20%–30% of infants versus 1% if acquired later in pregnancy.^2^, ^4^, ^5^

Congenital cytomegalovirus can lead to developmental delays, intrauterine growth restriction, microcephaly, prematurity, cerebral palsy, periventricular calcifications, epilepsy, hepatosplenomegaly, hepatitis, jaundice, chorioretinitis, and pneumonia.^1^, ^4^, ^5^ Neonates infected with cytomegalovirus and who are symptomatic may develop neurological impairments, including sensorineural hearing loss (most common), visual disturbances (such as blindness), and intellectual disability. Newborns suspected of having congenital cytomegalovirus may be tested for cytomegalovirus DNA in saliva, blood, or urine. Fetuses with a severe intrauterine cytomegalovirus infection might develop serious disorders affecting the brain, liver, spleen, and lungs, or they may experience spontaneous abortion, stillbirth, or postnatal death. Stillbirth (defined in the United States as fetal death at or after 20 weeks of gestation) occurs in about 1 in 175 pregnancies, with nearly 2 million stillbirths estimated globally each year. Death occurs in 4% of postnatal infants (during the first 6–8 weeks after birth) with severe congenital cytomegalovirus infection. Nearly 15% of newborns infected with cytomegalovirus may initially appear healthy but may, months or years later, show signs of congenital cytomegalovirus, including hearing loss, visual impairment, and balance difficulties.

In addition to the hematopoietic compartment, maternal periodontitis may serve as a significant source of fetal cytomegalovirus infection and related disorders. Periodontitis lesions^6^, ^7^ and symptomatic endodontic abscesses^8^, ^9^ can harbor hundreds of thousands of active (lytic) cytomegalovirus particles. The healthy, noninflamed periodontium shows no PCR‐detectable cytomegalovirus. Kumar et al.^10^ treated patients with aggressive periodontitis. After 3 months, they observed a 37% reduction in cytomegalovirus serum IgG antibodies, indicating that severe periodontitis lesions contain a substantial portion of the total body load of cytomegalovirus.

Herpesviruses typically enter the gingivitis sites as latent DNA found in the nuclei of monocytes/macrophages (cytomegalovirus, herpes simplex virus‐1), T‐lymphocytes (cytomegalovirus, herpes simplex virus‐1), and B‐lymphocytes/plasma cells (Epstein–Barr virus).^11^ Periodontal latent herpesviruses can activate in response to butyric acid,^12^ an extracellular metabolite produced by co‐resident *Porphyromonas gingivalis* and *Fusobacterium nucleatum*/*Fusobacterium periodonticum*, or to other bacterial products, pro‐inflammatory cytokines, and hormonal imbalances.^13^ Cytomegalovirus virions in the periodontium have easy access to the systemic circulation and may infect a fetus via transplacental transmission, especially in mothers with suppressed T‐lymphocyte functions. Hormonal changes during pregnancy lead to a systematic shift in mothers from T‐helper 1 (Th1) cell‐mediated responses (targeting herpesvirus‐infected cells) to T‐helper 2 (Th2) antibody‐promoting responses (preventing herpesvirus virions from entering healthy cells).^14^ Cytomegalovirus may be the most critical infectious agent for non‐genetic congenital diseases.^15^

Periodontal bacterial DNA can also inhabit human placental tissues. Bacterial DNA in the placenta most likely originates from maternal circulating DNA rather than live, virulent bacteria. Circulatory bacterial DNA counts may increase with oral hygiene neglect or secondary to a periodontal herpesvirus infection.^7^ Furthermore, the prevention and treatment of congenital diseases are poorly defined with inconsistent outcomes, necessitating new research approaches. The present article proposes an anti‐cytomegalovirus periodontal therapy for women with periodontitis and weakened immunity who are considering pregnancy or are in the first trimester of gestation.

## 2 OROFACIAL CLEFTS

Orofacial birth defects are among the most common physical disorders in the United States, with 1 in 700 newborns suffering from solely cleft palate or cleft lip with or without accompanying palate and alveolar clefts.^16^, ^17^ The lip is fully formed in week 5 post‐conception, the palate is formed between weeks 7 and 11, and all organs and body systems are developing by week 13 (end of the first trimester). These are timeframes within the high‐risk period for cytomegalovirus congenital infection and sequelae.^2^

Orofacial clefts can result from infectious, genetic, or environmental factors during embryonic development; however, the exact cause remains unclear in many cases. Weakened maternal innate defenses may increase the risk of fetal infections.^18^ Healthy immunocompetent women with preformed anti‐cytomegalovirus antibodies typically do not cause cytomegalovirus infection in unborn babies. Orofacial clefts can interfere with breast or bottle feeding, swallowing, breathing, hearing, and speech and language development. Most surgical repairs are performed within the first 6 months for cleft lip and within 12 months for cleft palate. Some children may require therapy into adulthood. Submucosal cleft palates and bifid uvulas may not be diagnosed at birth, and individuals born with orofacial clefts might eventually need speech therapy and prosthodontic or orthodontic treatment.

## 3 CYTOMEGALOVIRUS AND OROFACIAL BIRTH DEFECTS

Cytomegalovirus has been linked to the development of orofacial clefts.^15^ Divya et al.^19^ found among 6–14‐year‐old children that serum anti‐cytomegalovirus IgG antibodies were present in all 20 children with cleft lip/palate, in 20 completely deaf children, and 20 children with intellectual disabilities (indicating signs of congenital cytomegalovirus). In contrast, 20 healthy control children were either negative (16 children) or borderline positive (4 children) for cytomegalovirus IgG antibodies.^19^ Cytomegalovirus IgM antibodies were absent in 79 children and borderline positive in 1 child, suggesting no acute or exacerbated cytomegalovirus infection.^19^ Cytomegalovirus IgG with low avidity and elevated IgM antibodies are used diagnostically to identify a maternal primary infection within the preceding 3–4 months, which could result in congenital disease.^20^ Another serological study on orofacial clefts included infants aged 1 day to 10 months.^21^ Positive serum antibody titers against cytomegalovirus were detected in 64.5% of infants with orofacial clefts and 84.2% of their mothers, compared to 32.2% of control infants and 49.1% of their mothers (the difference between test and control infants, as well as between their mothers, was significant, *p* = 0.001).^21^ The cytomegalovirus immunologic type, the children's age, and the maternal immune status can affect serological findings.

Cytomegalovirus has recently been identified as the virus most closely associated with orofacial clefts by a wide margin.^22^ Various viruses, including other herpesvirus genera, hepatitis B virus, rubella virus, parvovirus, and HIV, may enhance the pathogenicity of cytomegalovirus or, to a lesser extent, contribute independently to the development of orofacial clefts.^21^, ^22^ Maternal periodontitis with unspecified viruses, but likely containing cytomegalovirus, has been linked to orofacial clefts,^23^ premature birth (before 37 weeks of pregnancy),^24^ low‐birth‐weight infants **(**under 2500 g),^24^ and preeclampsia.^25^ In a fetus at 29 weeks of gestation, cleft lip, micrognathia, and multiple signs of cytomegalovirus infection were detected by ultrasound examination.^26^ Cytomegalovirus may also cause missing teeth, fused teeth, or extra teeth in babies with orofacial clefts, but research data are pending. These studies collectively indicate that periodontal cytomegalovirus plays a significant role in the etiopathology of orofacial clefts and other congenital disorders.

Juvenile and prepubertal periodontitis may begin in the uterus. Cytomegalovirus infection of odontogenic cells can disrupt normal tooth development^27^, ^28^ and lead to structural defects in developing teeth,^29^ such as the cemental hypoplasia associated with juvenile periodontitis.^6^, ^30^ The cemental hypoplasia occurs within the bony socket of affected teeth, ruling out the periodontal pocket environment as a cause of the cemental defects.^30^ Histologic studies have shown that periodontal breakdown tends to start and progress in areas of cemental hypoplasia.^31^ The immune reactivation of periodontal cytomegalovirus or other herpesviruses at puberty produces pro‐inflammatory cytokines^32^, ^33^, ^34^ and an overgrowth of periodontopathic bacteria (*Aggregatibacter actinomycetemcomitans*, *Porphyromonas gingivalis*),^6^ triggering the rapid tissue destruction characteristic of juvenile periodontitis.

## 4 TREATMENT OF CONGENITAL CYTOMEGALOVIRUS

Valacyclovir/acyclovir, famciclovir, and valganciclovir are first‐choice drugs for herpes viruses. These anti‐viral medications, respectively, are prodrugs that convert in vivo to acyclovir, penciclovir, and ganciclovir to inhibit herpesvirus DNA polymerase. Valacyclovir is a low‐risk medicine widely prescribed in medical practice. Compared to oral acyclovir, valacyclovir shows increased absorption, bioavailability, and half‐life, requiring fewer daily applications. Valacyclovir's common side effects include headache, nausea, abdominal pain, fatigue, and confusion, which usually resolve quickly after discontinuing the drug. Valacyclovir treatment may precipitate acyclovir crystals in the renal tubules, causing a rare acute kidney injury, especially with preexisting kidney disease, valacyclovir overdosage, severe dehydration, elderly age, and nephrotoxic co‐medications (including common antimicrobials and analgesics). Valacyclovir and nephrotoxic drugs are preferably administered sequentially rather than concurrently.^35^ Famciclovir is indicated for treating infections caused by the herpes simplex virus and the varicella‐zoster virus. Famciclovir exhibits mild common side effects similar to those of valacyclovir. Valganciclovir/ganciclovir is used to treat HIV‐associated cytomegalovirus retinitis and to prevent organ transplant rejection. Valganciclovir may cause severe blood disorders, as well as cancer and birth defects in laboratory animals, and is usually not recommended for periodontal therapy. Drug treatment may exhibit unexpected adverse effects in some patients, which need to be weighed against inadequate periodontal therapy, where persisting periodontal pathogens (e.g., cytomegalovirus) can cause medical disorders or lead to tooth loss.

A recent evidence‐based therapy aimed to reduce mother‐to‐fetus transmission of cytomegalovirus. Shaha‐Nissan et al.^36^ demonstrated in 2020 that a high dosage of valacyclovir (8 g daily from enrollment until amniocentesis at 21/22 gestational weeks) decreased the rate of vertical cytomegalovirus transmission by 70% in women who had acquired a primary cytomegalovirus infection during the pre‐conception period or in the first trimester of pregnancy. Recent comprehensive reviews and clinical studies have supported the efficacy of valacyclovir in the secondary prevention of congenital cytomegalovirus.^37^, ^38^, ^39^, ^40^ Only 24 of 205 women experienced mild to moderate reversible symptoms from the extensive valacyclovir therapy, and no clinically significant adverse events were reported.^36^, ^40^

Periodontal treatment of pregnant women aims to reduce the risk of congenital diseases while ensuring a safe pregnancy. A systematic review, although only including a few studies, suggested that periodontal treatment decreased the incidence of preterm low‐birth‐weight babies by 57% and preterm births by 50%.^41^ The periodontal treatment suggested here focuses on neutralizing maternal active cytomegalovirus infection during the 2–3 months before conception and the high‐risk first trimester of pregnancy. Valacyclovir is the primary antiviral agent for periodontitis and other oral diseases involving cytomegalovirus, Epstein–Barr virus, or herpes simplex virus‐1. Valacyclovir used as a short‐term low‐dose monotherapy has resolved severe refractory periodontitis^42^ and acute endodontic abscesses.^43^ Despite several efforts, an effective vaccine for cytomegalovirus has yet to be developed.

In pre‐ or early pregnancy, periodontal therapy involves a single treatment with valacyclovir (1 g BID on days 1 and 2, and 500 mg BID on days 3–7) and subgingival and supragingival ultrasonic scaling with 0.1%–0.2% sodium hypochlorite cooling fluid but omits anaerobic antibiotics and periodontal surgery. PCR quantification of cytomegalovirus in maternal saliva^44^ or periodontal pockets^45^ may monitor the therapeutic effectiveness. Well‐qualified dentists (periodontists) ought to perform the periodontal diagnosis and the antiviral drug therapy.

Patient self‐care includes individualized oral hygiene instruction, daily subgingival irrigation with freshly prepared 0.1%–0.2% sodium hypochlorite using the Waterpik Pik Pocket or a similar delivery device, and twice weekly oral rinsing for 30 seconds with 0.1%–0.2% sodium hypochlorite. Sodium hypochlorite occurs naturally in monocytes/macrophages and neutrophils and is a safe^46^, ^47^ and effective^48^, ^49^ antimicrobial mouth rinse available as medical grade or regular household bleach. Sodium hypochlorite is used prophylactically in food safety, water treatment, hospital disinfection, and therapeutically in vivo for wound antiseptics and nasopharynx infections. A 0.15% sodium hypochlorite oral rinsing solution can be obtained by mixing 1 part of Clorox regular bleach (7.5%) with 50 parts of water. The hypochlorite oral rinses tend to eliminate major periodontopathic bacteria in supragingival and subgingival biofilms.^50^ In addition to potentially preventing congenital cytomegalovirus in the baby, the combined valacyclovir/sodium hypochlorite treatment will protect the pregnant mother against tooth loss from herpesviruses and major periodontopathic bacteria. Postdelivery, the mother may need additional periodontal treatment to address the periodontitis‐affected dentition. Also, a woman having a child with congenital cytomegalovirus may receive periodontal treatment to prevent congenital disorders in future pregnancies.

## 5 SUMMARY AND CONCLUSIONS

Contemporary periodontology emphasizes preventing tooth loss and reducing the risks of focal infections.^51^, ^52^, ^53^ Cytomegalovirus has been linked to cleft lip and cleft palate, yet the source of this virus remains an enigma. Maternal periodontitis can contain high copy counts of active cytomegalovirus,^54^, ^55^ presumably representing a major nidus for developing orofacial clefts. This paper proposes an anti‐cytomegalovirus periodontal therapy during the preconception period and the first trimester of pregnancy to reduce cytomegalovirus fetal infection and its sequelae. In addition to neutralizing periodontal cytomegalovirus and reducing orofacial cleft risks, the periodontal treatment improves the periodontal health status and decreases the risk of tooth loss during pregnancy. Treating active periodontal cytomegalovirus is conceptually and therapeutically straightforward. Systemic and topical anti‐herpesvirus therapy can neutralize active cytomegalovirus in the periodontium, after which the cytomegalovirus enters the latency phase. The patient's diligent anti‐gingivitis efforts help prevent latent cytomegalovirus embedded in inflammatory cells from infiltrating the periodontium. Pregnant women typically follow treatment recommendations to support the healthy development of the fetus.^36^ Still, most pregnant women are unaware that periodontal disease can lead to severe birth defects and do not seek dental advice during their pregnancy.^56^ Public education and outreach programs in oral healthcare are essential to raising awareness about the risks of fetal disorders caused by untreated maternal periodontitis.^57^ Research on the role of periodontal cytomegalovirus and other oral causes of birth defects is strongly encouraged.^15^, ^58^, ^59^ In summary, cytomegalovirus in maternal periodontitis is likely a critical nidus for fetal infection. This study identifies high‐risk individuals, affordable treatment options, and a clear therapeutic endpoint to help control congenital diseases.

## 6 COMMUNICATING WITH LAYPERSONS

Periodontology is the dental field that focuses on preventing and treating gum disease to avoid loose teeth and tooth loss. Research shows that the bacteria and viruses responsible for gum disease can enter the blood vessels surrounding the teeth and circulate throughout the body, increasing the risk of diseases in various tissues and organs. Deep pockets between the gums and teeth are a sign of severe gum disease. Diseased gums may contain hundreds of thousands of active cytomegaloviruses, while healthy gums contain no cytomegalovirus. Cytomegalovirus is a member of the herpes virus family. Normal daily activities, such as chewing and tooth brushing, can push the cytomegalovirus from a mother with severe gum disease into the bloodstream, posing a risk of infecting an unborn baby. Mothers with weak immune systems are at the highest risk of causing an infection in the unborn baby. The disease condition known as Congenital Cytomegalovirus may lead to serious birth defects, including cleft lip and palate, as well as stillbirth. Approximately 5500 infants are born each year in the USA with congenital cytomegalovirus and related health issues at birth or later in life. To protect the unborn baby, mothers with gum disease need professional treatment to transform their diseased gums into healthy, cytomegalovirus‐free gums. The treatment involves a common antiviral medication taken for one week, daily specialized oral hygiene techniques, and regular oral hygiene at home. The specialized oral hygiene period extends from two months before pregnancy to the end of the first trimester. The recommended treatment is affordable, painless, and highly safe for the pregnant mother and the unborn baby.

## CONFLICT OF INTEREST STATEMENT

The author declares no conflict of interest.

## DISCLOSURE

Publication cost is covered by the University of Southern California, according to an agreement between Wiley and a group of California universities: Statewide California Electronic Library Consortium (SCELC) Member Institutions.

## Data Availability

No new data were created or analyzed in this study. Data sharing is not applicable to this article.
